# Sulforaphane Prevents Hepatic Insulin Resistance by Blocking Serine Palmitoyltransferase 3-Mediated Ceramide Biosynthesis

**DOI:** 10.3390/nu11051185

**Published:** 2019-05-27

**Authors:** Wendi Teng, Yuan Li, Min Du, Xingen Lei, Siyu Xie, Fazheng Ren

**Affiliations:** 1 Beijing Advanced Innovation Center for Food Nutrition and Human Health, College of Food Science & Nutritional Engineering, China Agricultural University, Beijing 100083, China; tengwendidi@163.com (W.T.); xiesiyu2406@163.com (S.X.); 2 Key Laboratory of Functional Dairy, Co-constructed by ministry of Education and Beijing Municipality, College of Food Science & Nutritional Engineering, China Agricultural University, Beijing 100083, China; yuanli@cau.edu.cn; 3 Department of Animal Sciences, Washington State University, Pullman, WA 99164, USA; min.du@wsu.edu; 4 Department of Animal Science, Cornell University, Ithaca, NY 14853, USA; xl20@cornell.edu

**Keywords:** sulforaphane, insulin resistance, glucose uptake, ceramide, liver

## Abstract

Sulforaphane (SFA), a naturally active isothiocyanate compound from cruciferous vegetables used in clinical trials for cancer treatment, was found to possess potency to alleviate insulin resistance. But its underlying molecular mechanisms are still incompletely understood. In this study, we assessed whether SFA could improve insulin sensitivity and glucose homeostasis both in vitro and in vivo by regulating ceramide production. The effects of SFA on glucose metabolism and expression levels of key proteins in the hepatic insulin signaling pathway were evaluated in insulin-resistant human hepatic carcinoma HepG2 cells. The results showed that SFA dose-dependently increased glucose uptake and intracellular glycogen content by regulating the insulin receptor substrate 1 (IRS-1)/protein kinase B (Akt) signaling pathway in insulin-resistant HepG2 cells. SFA also reduced ceramide contents and downregulated transcription of ceramide-related genes. In addition, knockdown of serine palmitoyltransferase 3 (SPTLC3) in HepG2 cells prevented ceramide accumulation and alleviated insulin resistance. Moreover, SFA treatment improved glucose tolerance and insulin sensitivity, inhibited SPTLC3 expression and hepatic ceramide production and reduced hepatic triglyceride content in vivo. We conclude that SFA recovers glucose homeostasis and improves insulin sensitivity by blocking ceramide biosynthesis through modulating SPTLC3, indicating that SFA may be a potential candidate for prevention and amelioration of hepatic insulin resistance via a ceramide-dependent mechanism.

## 1. Introduction

Type 2 diabetes mellitus (T2DM), an intricate metabolic disorder, has emerged as a major global public health problem [1]. According to the estimates of the World Health Organization, its global occurrence rate in 2016 was, strikingly, at 8.5%, meaning about 1 in 12 people suffer from this disease [2]. The morbidity and mortality due to T2DM are very high because of its close association with numerous diabetes-related complications, such as retinopathy, blindness and nephropathy [3,4,5]. Insulin resistance is one of the hallmarks of T2DM. The liver is an organ deeply implicated in insulin resistance [6]. Indeed, ectopic lipid accumulation in liver and peripheral tissues due to obesity results in insulin resistance, a key risk factor for developing T2DM [7,8]. In humans, higher concentrations of free fatty acids in plasma induce insulin desensitization [9]. Saturated fatty acids, in particular, inhibit insulin signal transduction by activating a variety of kinases [10] and subsequently result in decreased glucose uptake and glycogen synthesis, as well as peripheral insulin resistance [11]. 

In the process, hepatic ceramide levels closely correlate with hepatic insulin resistance. Wistar rats fed on 5-week high fat diet (HFD) displayed increased total hepatic ceramides in concert with elevated index of homeostatic model assessment of insulin resistance (HOMA-IR) [12]. Leptin-deficient ob/ob mice had higher total hepatic ceramides [13]. Ceramide is synthesized initially from palmitate and serine via the action of serine palmitoyltransferase (SPTLC) [14]. SPTLC converts serine and palmitoyl-CoA into 3-ketosphinganine, which is sequentially catalyzed by 3-ketosphinganine reductase, dihydroceramide synthase (CerS) and dihydroceramide desaturase (DEGS1) to form ceramide [15]. Inhibitors of SPTLC and CerS, which prevent ceramide accumulation, have potent insulin-sensitizing effects both in vitro and in vivo [16,17]. Due to the important role of ceramide in the progression of obesity-related metabolic dysfunction, reducing ceramide content, either pharmacologically or genetically, might be a novel approach to preventing or treating obesity-induced insulin resistance and T2DM [15,18].

Sulforaphane (SFA) is a naturally-occurring isothiocyanate compound isolated from cruciferous vegetables such as broccoli and cabbage. Originally used for anticancer therapy [19,20], SFA has recently been shown to have antidiabetic effects in mice and obese patients [21,22]. Annika S. Axelsson et al. reported SFA could reduce fasting blood glucose and glycated hemoglobin in obese patients with dysregulated type 2 diabetes [21]. Naoto Nagata showed glucoraphanin, a stable glucosinolate precursor of SFA, ameliorated obesity and insulin resistance through adipose tissue browning in mice [22]. Clinical trials are currently underway investigating the utility of this compound in controlling blood glucose level and insulin resistance. Although nuclear factor erythroid-related factor 2 (Nrf2) was suggested as a target of SFA to explain its antidiabetic effects [22], recent evidence suggested that SFA might have additional actions relevant to metabolic diseases [23,24]. The purpose of this study was to assess the effects of SFA on alleviating insulin resistance and investigate the hypothesis that SFA could ameliorate insulin resistance by blocking ceramide biosynthesis both in vitro and in vivo.

## 2. Materials and Methods

### 2.1. Materials and Chemicals

SFA (purity > 99%) was kindly provided by LKT Laboratories (St. Paul, MN, USA). Palmitic acid (PA) and human recombinant insulin were from Sigma-Aldrich, USA. TRIzol, lipofectamine 2000, and 2-[N-(7-nitrobenz-2-oxa1,3-diazol-4-yl) amino]-2-deoxy-D-glucose (2-NBDG) were from Life Technologies (Carlsbad, CA, USA). Bicinchoninic acid protein concentration determination kit (BCA kit), triglycerides (TG) assay kit, total cholesterol (TC) assay kit, alanine aminotransferase (ALT) assay kit and aspartate aminotransferase (AST) assay kit were from Nanjing Jiancheng Bioengineering Institute, Nanjing, China. All chemicals used in the study were of analytical grade.

### 2.2. Cell Culture and Establishment of Insulin-Resistant Cell Model

Human hepatic carcinoma HepG2 cells obtained from Beijing Union Medical Cell Resource Center (Basic Medical Cell Center, Institute of Basic Medical Sciences, Chinese Academy of Medical Sciences) were cultured in minimum essential medium (MEM) containing 10% fetal bovine serum, 1% non-essential amino acids, 100 µg/mL streptomycin and 100 U/mL penicillin (Invitrogen, Carlsbad, CA, USA) at 37 °C in an incubator with 5% CO_2_ and 95% humidity. 

An insulin-resistant cell model was established according to the previously described method [25]. Briefly, 5 × 10^4^ HepG2 cells were seeded into each well of 12-well plates. After 12 h incubation, the medium was changed to MEM containing 0, 25, 50, 100, 150 and 200 µM PA for 12, 24 and 36 h followed by 100 nM insulin stimulation for 10 min. Based on cellular response to insulin stimulation, 100 µM of PA for 24 h were selected for establishing insulin-resistant HepG2 cells.

### 2.3. Cell Viability Assessment

The viability of HepG2 cells was analyzed using cell counting kit-8 (CCK-8) colorimetric assay (Beyotime, Haimen, Jiangsu, China) according to the manufacturer’s instructions. [2-(2-methoxy-4-nitrophenyl)-3-(4-nitrophenyl)-5-(2,4-disulfophenyl)-2H-tetrazolium, monosodium salt] (WST-8) was reduced by dehydrogenases in cells to give an orange-colored formation dye, which is soluble in the culture medium. The amount of the formation dye generated by dehydrogenases is directly proportional to the number of living cells. In brief, HepG2 cells were seeded into 96-well plates at a density of 1 × 10^4^ cells per well. After treatments, CCK8 reagent was added to each well, and cells were incubated at 37 °C in a 5% CO_2_ incubator for another 1 h. Then, the absorbance at 450 nm was quantified using a microplate reader (Bio-Rad, Hercules, CA, USA), and cell viability was calculated as percentage values, as compared to the control group.

### 2.4. Cellular Glucose Uptake Analysis

2-NBDG is a fluorescent glucose analog that has been used to monitor glucose uptake in live cells. Thus, in this study we used 2-NBDG reagent to detect cellular glucose uptake, as described previously [26]. Briefly, cells were plated into 12-well plates at a density of 5 × 10^4^ cells per well. After treatments, cells were washed twice with PBS and mixed with 10 µM 2-NBDG solution at 37 °C. After 1 h, cells were again washed three times with PBS to remove remaining 2-NBDG reagent, and subsequently suspended for flow cytometry analysis using Fluorescence Activated Cell Sorter (FACS) CytoFLEX flow cytometer (Beckman Coulter, S. Kraemer Boulevard Brea, CA, USA) at an excitation wavelength of 488 nm and an emission wavelength of 535 nm. Data were analyzed using CytExpert 2.0.0 software (Beckman Coulter, S. Kraemer Boulevard Brea, CA, USA).

### 2.5. Determination of Intracellular Glycogen Content

To measure glycogen content, cells were seeded into a 6-well plate at a density of 3 × 10^5^ cells per well. After treatment for 24 h, cells were washed three times with PBS and collected. The glycogen content in the cells was assayed using anthrone reagent (Solarbio, Beijing, China), and the amount of blue compound generated by this reaction was detected at 620 nm using a microplate reader (Bio-Rad, Hercules, CA, USA). In addition, the protein content of the collected HepG2 cells was quantified using the BCA method and the glycogen level was expressed as the ratio of glycogen (mg)/protein (mg).

### 2.6. Animals and Diets

Male C57BL/6J mice (6 weeks old) from Beijing Vital River Laboratory Animal Technology Co (Beijing, China) were housed in a room with a controlled environment (temperature, 22–24 °C; humidity, 60%) under a 12 h light-dark cycle. After 1 week of acclimatization, the mice were randomly divided into four groups (n = 10 per group) as follows: normal chow (NC; 12450b, Research Diets, NJ, USA, 10% kcal fat content), high fat diet (HFD; D12492, Research Diets, NJ, USA, 60% kcal fat content), HFD with 0.5 mg/kg SFA (three times a week, i.p.), and HFD with 5 mg/kg SFA (three times a week, i.p.). Body weight was recorded weekly. After 10 weeks, the mice were deprived of food overnight and anesthetized. The blood samples were taken from the vena cava. The liver was immediately excised, weighed, and stored at −80 °C. All procedures were performed in accordance with the Guide for the Care and Use of Laboratory Animals and were approved by the Experimental Animal Ethics Committee at China Agricultural University. 

### 2.7. Body Fat Distribution and Content

The body fat distribution and content in mice (n = 6) were measured under anesthesia with 4% chloral hydrate (4 mL/kg) using MesoMR23-060H-I imaging instrument (Shanghai Niumag Corporation, Shanghai, China) at parameters setting as K space = 192 × 256 mm, time of echo = 13.5 ms, time of waiting = 300 ms, magnet= 0.55 T, section thickness = 3.5 mm, field of view read =100 mm, field of view phase = 100 mm and number of scans = 8.

### 2.8. Hematoxylin and Eosin as well as Oil Red O Staining

Liver tissues were fixed with 10% formalin for 10 min and prepared as 5–7 µm sections. Oil Red O working solution was prepared by mixing six parts of 0.5% Oil Red O isopropanol stock solution with 4 parts of water. The sections were stained with Oil Red O working solution for 1 h, counterstained with hematoxylin and eosin for 15 min, washed twice with water, mounted on slides with glycerin jelly (Burlington, NC, USA), and observed and photographed under a microscope.

### 2.9. Biochemical Analyses of Liver Tissues

Liver tissues were homogenized in 4 volumes of ice-cold 50 mM Tris-HCl buffer (pH 7.4) containing 1 mM ethylenediaminetetraacetic acid (EDTA), and centrifuged at 12,000× *g* for 30 min at 4 °C. The supernatants were used to analyze the contents of TG, TC, ALT and AST in the liver using biochemical assay kits, according to the manufacturer’s instructions.

### 2.10. Glucose Tolerance Test and Insulin Tolerance Test

For glucose tolerance test, mice were intraperitoneally injected with glucose at 2 g per kg body weight after 12 h of fasting. For insulin tolerance test, mice were intraperitoneally injected with insulin (Eli Lilly and Company, Indianapolis, IN, USA) at 1 IU per kg body weight after 6 h of fasting. In all tests, tail blood glucose levels were measured with a glucometer (Abbott, Chicago, IL, USA) at the indicated times (0, 30, 60, 90, 120 and 180 min) after injection.

### 2.11. Ceramide Content Analysis

After treatment, HepG2 cell suspension and liver homogenate were collected. After protein measurement, the samples were fortified with 50 µL internal standard solutions and used for lipids extraction using 2 mL of extraction mixture (iso-propanol: water: ethyl acetate = 30:10:60; v: v: v). The samples were vortexed, sonicated 30 s for 3 times and centrifuged for 10 min at 4000 rpm. The supernatants were transferred into a new vial and re-extracted as described above. The extracted supernatants were dried under a gentle nitrogen stream and subjected to quantification of ceramides on an Agilent high performance liquid chromatography system coupled with a quadrupole-time of flight mass spectrometer (6545 Q-TOF), as described previously [27,28], based on the C17-ceramide internal standards from Avanti Polar Lipids (Alabaster, AL, USA) as well as C14-ceramide, C18-ceramide, C20-ceramide, C22-ceramide, C24-ceramide, C24:1-ceramide and C26:1-ceramide standards from ZZStandard (Shanghai, China). Ceramide level is presented as µmol/protein (mg/mL).

### 2.12. RNA Interference

Small interfering RNAs (siRNA) targeting SPTLC3, CerS2 and CerS4 (Supplementary Table S1) to knockdown their expression as well as a scramble siRNA were transfected into 70% confluence HepG2 cells using lipofectamine 2000 (Life Technologies, Carlsbad, CA, USA) according to the manufacturer’s instructions. At 24 h after transfection, cells were treated as needed and collected for analysis.

### 2.13. Real-Time PCR Analysis

Total RNA was extracted from HepG2 cells and liver tissues using TRIzol reagent and used for Real-time quantitative polymerase chain reaction (RT-PCR) analysis using a SYBR green-based kit according to the manufacturer’s instructions on a 7900HT instrument (Applied Biosystems, Forster, CA, USA). The specificity of PCR products was evaluated using the melting curve analysis. Relative gene expression was determined using the 2-∆∆ method. Primers used for RT-PCR were shown in Supplementary Table S2.

### 2.14. Western Blotting

Western blots were performed as described previously [29] for glycogen synthase kinase 3 beta (GSK3β; Abcam, Cambridge, UK), phospho-GSK3β Ser9 (Abcam), glycogen synthase 1 (GS; Abcam), phospho-GS Ser641 (Abcam), β-actin (Abcam), protein kinase B (Akt; Cell Signaling Technology, Beverly, MA, USA), phospho-Akt Ser473 (CST), insulin receptor substrate 1 (IRS-1; CST), phospho-IRS-1 Ser307 (CST), phospho-IRS-1 Y632 (CST), forkhead box protein O1 (FoxO1; CST) and phospho-FoxO1 Thr24 (CST) [30,31].

### 2.15. Statistical Analysis

Data are expressed as mean ± standard deviation (SD) for at least three independent experiments. Data were analyzed with SPSS statistical software (version 21.0, IBM, Chicago, IL, USA) using one-way analysis of variance (ANOVA) followed by the Duncan’s test, with the threshold for significance being *p* < 0.05.

## 3. Results

### 3.1. SFA Improved Glucose Uptake and Modulated Insulin Signaling Pathway in Insulin-Resistant HepG2 Cells

**Figure 1:**
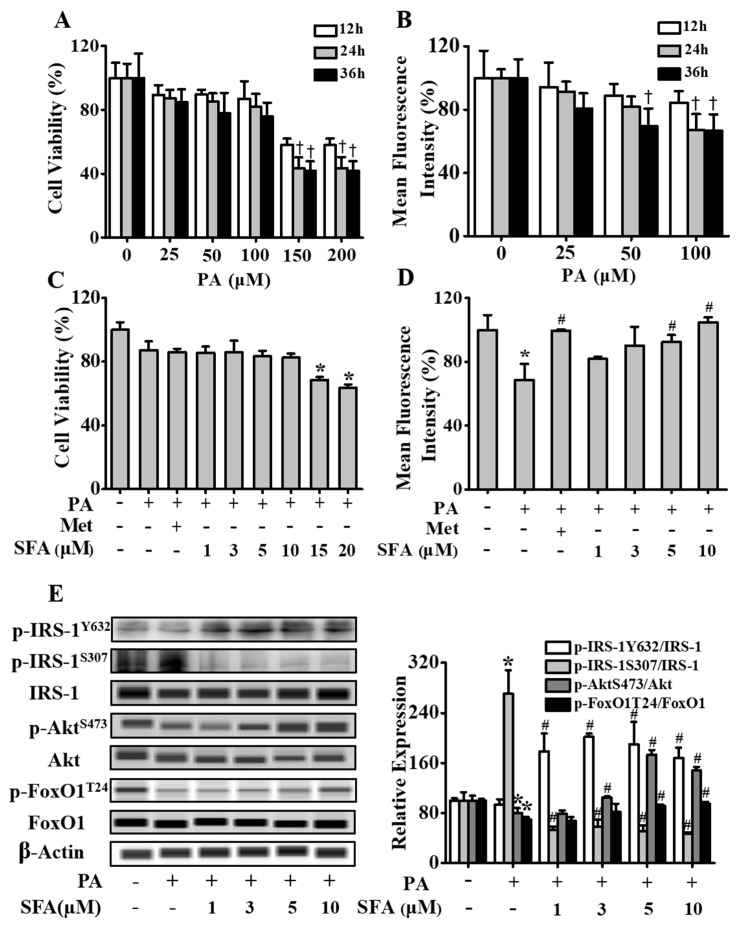
Effects of sulforaphane (SFA) on cell viability, glucose uptake and expression of key proteins in insulin signaling pathway in insulin-resistant HepG2 cells. (**A**) HepG2 cells treated with palmitic acid (PA) at different concentrations (0, 25, 50, 100, 150 and 200 µM) for 12, 24 and 36 h, respectively, followed by 100 nM insulin stimulation for 10 min, as measured using a cell counting kit-8 (CCK-8). Cell viability expressed as the percentage of viable cells relative to the total cells in the control group. (**B**) Cellular glucose uptake expressed as the percentage of mean fluorescence intensity relative to the control cells. (**C**) HepG2 cells treated with SFA at different concentrations (1, 3, 5, 10, 15 and 20 µM) or 250 µM of metformin in the presence of 100 µM PA for 24 h followed by 100 nM insulin treatment for 10 min. Cell viability expressed as the percentage of viable cells relative to the total cells in the control group. (**D**) Cellular glucose uptake expressed as the percentage of mean fluorescence intensity relative to the control cells. (**E**) Western blot analysis showing the levels of total and phosphorylated insulin receptor substrate 1 (IRS-1), protein kinase B (Akt) and forkhead box protein O1 (FoxO1) in HepG2 cells. And densitometric assay of each band. Representative western blot for phosphorylated and total levels of IRS-1, Akt and FoxO1 in HepG2 cells in the left side of panel of E. Fold change in optical density relative to controls in the right side of panel of E. Values shown are means ± standard deviation (SD), † *p* < 0.05 vs. different concentrations PA-treated cells at 12 h; * *p* < 0.05 vs. control cells; # *p* < 0.05 vs. only insulin-resistant HepG2 cells. PA, palmitic acid; SFA, sulforaphane; Met, metformin.

In order to develop a model of insulin resistance induced by PA in hepatic cells, HepG2 cells were first exposed to rising doses of PA (0, 25, 50, 100, 150 and 200 µM) for 12, 24 or 36 h followed by 100 nM insulin stimulation for 10 min and cell viability were evaluated. As shown in Figure 1A, compared with control group, cell viability was reduced to about 80% after treatment with 25, 50 or 100 µM PA, but there was no significance. Cell viability was significantly reduced after treatment with 150 µM and 200 µM PA. Thus, PA at 0, 25, 50 and 100 µM was used for glucose uptake test. As shown in Figure 1B, glucose uptake was decreased after treatment of HepG2 cells with PA at different concentrations, which might be a combined effects of cell viability and glucose uptake. In particular, when cells were incubated with 100 µM PA, cellular glucose uptake no longer dropped at 36h, suggesting the model remained stable at least for 24 h. Thus, 100 µM PA treatment for 24 h was used for further analyses.

To understand the potential roles of SFA in ameliorating PA-induced insulin resistance in HepG2 cells, we first measured its effect on cell viability. As shown in Figure 1C, SFA treatment higher than 10 µM significantly reduced cell viability. Therefore, 1, 3, 5 and 10 µM SFA treatments were used in the following experiments. As shown in Figure 1D, compared with control group, PA treatment significantly decreased cellular glucose uptake (*p* < 0.05). However, this decrease was alleviated by SFA in a dose-dependent manner. In addition, 10 µM SFA treatment showed nearly the same effect as 250 µM metformin did. We further analyzed the effects of SFA treatment on the level of total and phosphorylated key proteins in the insulin signaling pathway in insulin-resistant HepG2 cells. As shown in Figure 1E, 100 µM PA treatment stimulated phosphorylation of IRS-1 at Ser307. However, this stimulation was alleviated by SFA treatment. Meanwhile, treatment of HepG2 cells with 100 µM PA decreased phosphorylation of IRS-1 at tyrosine 632, Akt at serine 473 and FoxO1 at threonine 24. But SFA treatment rescued these reductions in a dose-dependent manner (Figure 1E). These results suggested that SFA treatment improved glucose uptake and modulated the insulin signaling pathway in PA-induced insulin-resistant HepG2 cells.

### 3.2. SFA Regulated Phosphorylation Levels of Key Glycogenic Proteins and Increased Intracellular Glycogen in Insulin-Resistant HepG2 Cells

**Figure 2:**
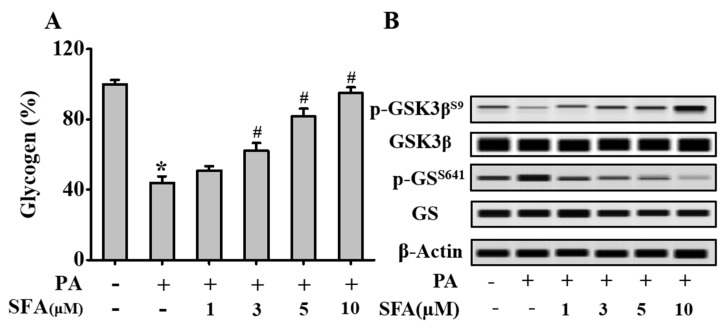
Effects of SFA treatment on the levels of phosphorylated glycogen synthase kinase 3 beta (GSK3β) and glycogen synthase 1 (GS), and intracellular glycogen in insulin-resistant HepG2 cells. HepG2 cells were treated with SFA at different concentrations (1, 3, 5 and 10 µM) in the presence of 100 µM PA for 24 h followed by 100 nM insulin treatment for 10 min. (**A**) Relative glycogen content in comparison to that of the control cells. (**B**) Western blot analysis showing the levels of phosphorylated GSK3β and GS in HepG2 cells. Data are means ± SD, * *p* < 0.05 vs. control cells; # *p* < 0.05 vs. only insulin-resistant HepG2 cells.

To investigate the effects of SFA on glycogenesis of insulin-resistant HepG2 cells, the levels of phosphorylated GSK3β, phosphorylated GS and intracellular glycogen were determined. Figure 2B showed that PA treatment remarkably lowered the level of phosphorylated GSK3β and this reduction was significantly reversed by treatment with SFA. On the other hand, PA treatment elevated the level of phosphorylated GS, but SFA treatment reversed this elevation. In addition, compared with the control group, PA treatment decreased the glycogen level (*p* < 0.05). However, the reduction of intracellular glycogen was recovered by SFA treatment in a concentration-dependent manner. (Figure 2A). Overall, these results showed that SFA increased intracellular glycogen by regulating phosphorylation levels of key proteins of glycogen synthesis in insulin-resistant HepG2 cells.

### 3.3. SFA Inhibited Ceramide Accumulation and Modulated Ceramide Biosynthesis in Insulin-Resistant HepG2 Cells

**Figure 3:**
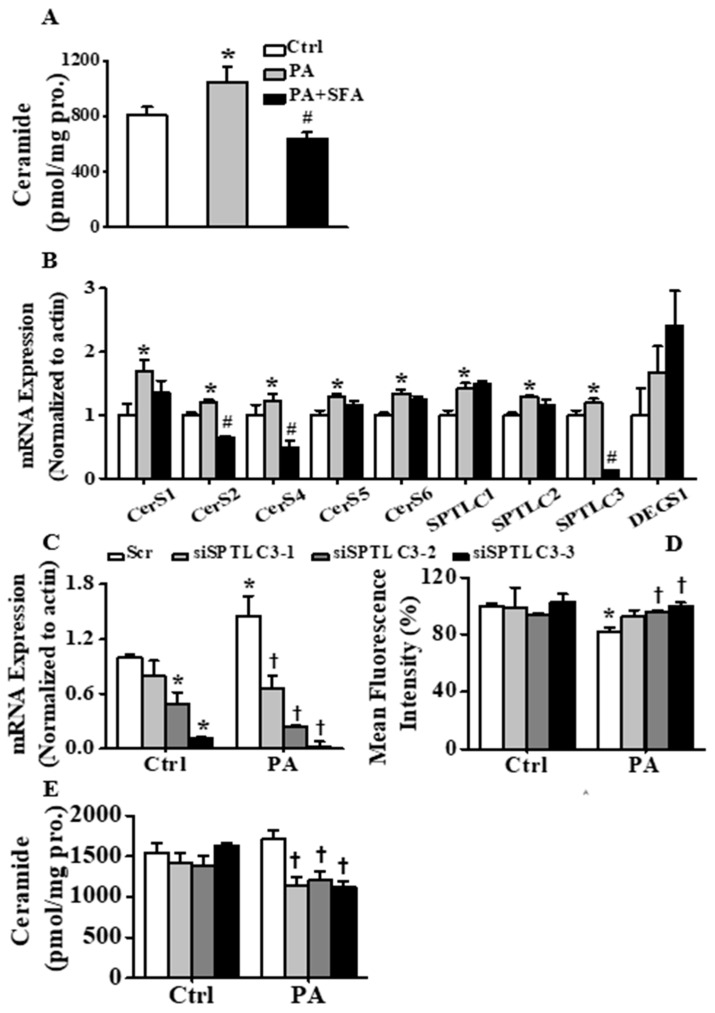
Effects of SFA on ceramide contents and transcription level of ceramide-related genes and effect of siSPTLC3 on glucose uptake in insulin-resistant HepG2 cells. (**A**) Total ceramide contents detected by LC/MS/MS and (**B**) transcription levels of serine palmitoyltransferase 1 (SPTLC1), 2 and 3, CerS1, 2, 4, 5 and 6, and dihydroceramide desaturase (DEGS1) determined by RT-PCR in HepG2 cells treated with or without 100 µM PA in the absence or presence of 10 µM SFA for 24 h, followed by 100 nM insulin stimulation for 10 min. (**C**) The transcription level of serine palmitoyltransferase 3 (SPTLC3) determined by RT-PCR, (**D**) cellular glucose uptake expressed as percentage of mean fluorescence intensity relative to control cells and (**E**) total ceramide contents detected by LC/MS/MS in HepG2 cells transfected with control small interfering RNAs (siRNA) or SPTLC3 siRNA and incubated in medium containing either normal or 100 µM PA for 24 h followed by 100 nM insulin stimulation for 10 min. All values are presented as means ± SD from three independent determinations. * *p* < 0.05 vs. control cells; # *p* < 0.05 vs. PA-treated cells; † *p* < 0.05 vs scramble group in PA-treated cells.

Several lines of evidence support a role for ceramides in the pathogenesis of insulin resistance. Intracellular ceramides act as “second messengers” to coordinate cellular responses to nutrients (e.g., saturated fatty acids) [32]. Therefore, we analyzed ceramide production in different conditions using liquid chromatograph tandem mass spectrometry (LC/MS/MS). Figure 3A and Figure S1 showed that PA treatment remarkably increased cellular ceramides levels (*p* < 0.05). Compared with PA group, treatment with SFA significantly reversed these decreases (*p* < 0.05). Furthermore, RT-PCR analysis (Figure 3B) of nine ceramide-related genes, including SPTLC1, 2 and 3, CerS1, 2, 4, 5 and 6, and DEGS1 revealed that PA treatment increased transcription of the above-mentioned genes. However, SFA treatment obviously reversed the enhancements of SPTLC3, CerS2 and CerS4 expression. Overall, these data strongly demonstrated that SFA reduced ceramides accumulation and regulated ceramide biosynthesis in insulin-resistant HepG2 cells. 

### 3.4. SFA Alleviated Insulin Resistance by Inhibiting Ceramide Formation in HepG2 Cells

Having confirmed the efficacy of SFA in preventing lipid-induced insulin resistance, we further explored the possible influence of ceramide biosynthesis on the insulin- sensitizing effects of SFA. siRNA targeting to SPTLC3, CerS2 and CerS4 were first transfected into HepG2 cells to correspondingly lower their mRNA levels (Figure 3C, Figure S2A,D). Interestingly, downregulation of SPTLC3 (Figure 3D) but not CerS2 and CerS4 (Figure S2B,E) partially reversed PA-induced reduction of cellular glucose uptake, indicating that SFA negated PA-induced insulin resistance in HepG2 cells by modulating SPTLC3. Moreover, SPTLC3 knockdown (Figure 3E and Figure S3) but not CerS2 and CerS4 knockdown (Figure S2C,F) prevented PA-induced ceramide increase in HepG2 cells, suggesting that SFA ameliorated insulin resistance through inhibiting ceramide biosynthesis by modulating SPTLC3 expression.

### 3.5. SFA Improved Insulin Sensitivity and Altered Ceramide Biosynthesis in HFD-Fed Mice

**Figure 4:**
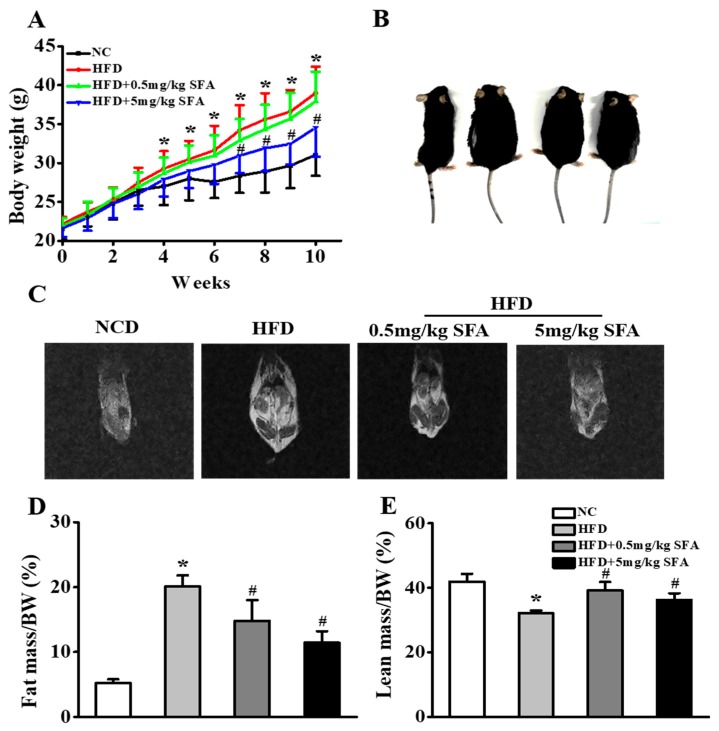
Inhibition effects of SFA treatment on body weight and fat distribution of high fat diet (HFD)-fed mice. (**A**) Body weight (n = 10/group). (**B**) Pictures of HFD-fed mice after 10 weeks of SFA intervention. (**C**) Magnetic Resonance Imaging (MRI) images showing fat distribution in mice. (**D**) Fat mass and (**E**) lean mass distribution (n = 6/group). Values shown are means ± SD, * *p* < 0.05 vs. normal chow (NC) group; # *p* < 0.05 vs. HFD group. NC, normal chow; HFD, high fat diet.

With evidence supporting the effect of SFA on alleviating insulin resistance in HepG2 cells, we sought to determine the effect of SFA on insulin sensitivity, metabolic parameters and ceramide biosynthesis in a mouse model of obesity and insulin resistance. We first examined the effects of SFA on mouse body weight and fat mass. After 10 weeks of administration, 5 mg/kg SFA group had a lower mouse body weight gain compared with HFD group (Figure 4A,B). This was largely attributed to the lower fat mass and higher lean mass (Figure 4C–E). Compared with the control mice, liver tissues of HFD-fed mice had yellow necrotic foci and grey-red color, lost skin luster and became tumescent. Surprisingly, SFA treatment avoided these changes (Figure S4Aa) and alleviated HFD-induced liver damage (Figure S4Ab). Particularly, Oil Red O staining of histological liver sections showed that SFA treatment significantly lowered lipid deposition in the liver compared to the HFD group (Figure S4Ac). Meanwhile, SFA treatment significantly lowered hepatic levels of TG, TC, ALT and AST in HFD-fed mice, and these attenuations were more dramatic in mice given 5 mg/kg SFA (Figure S4B–E). These data indicated that HFD successfully induced obesity and hepatic steatosis and SFA alleviated these changes. 

**Figure 5:**
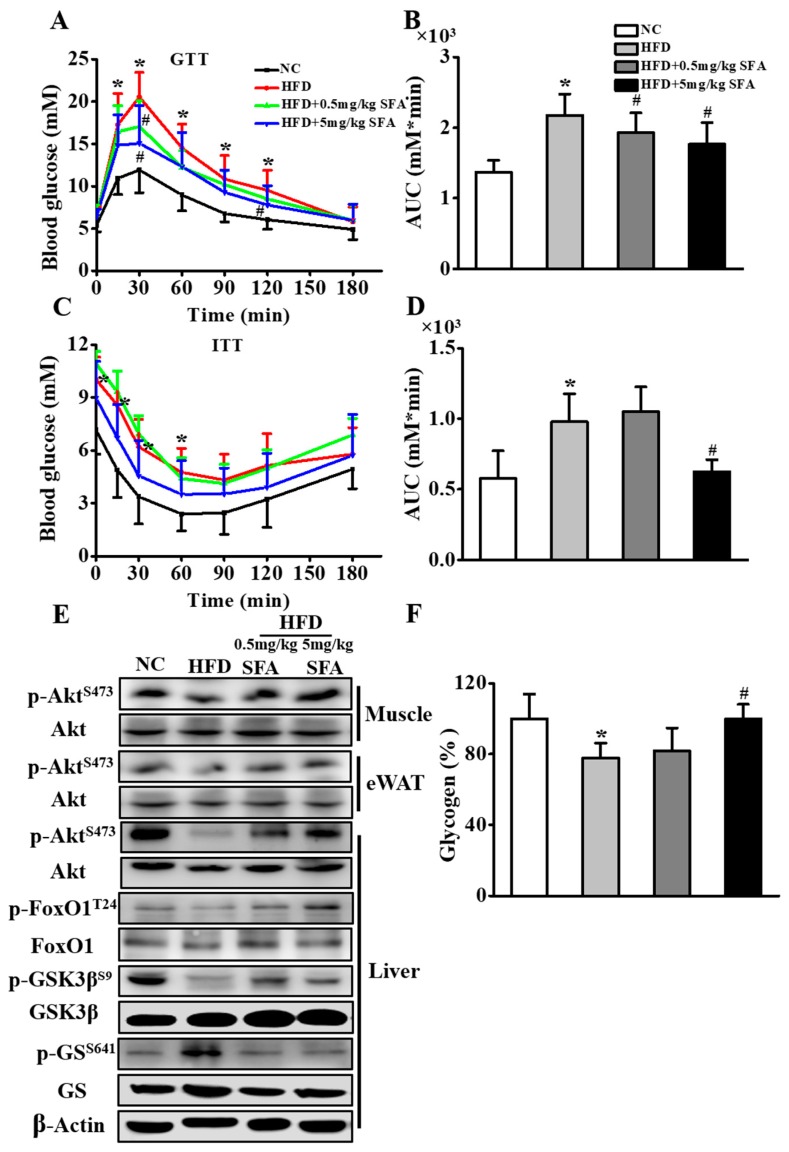
Enhanced inhibition effects of SFA on insulin resistance in vivo. (**A**) Glucose tolerance test (GTT) and (**B**) the area under the curve (AUC) of mice at week 10 (n = 10/group). (**C**) Insulin tolerance test (ITT) and (**D**) AUC of mice at week 10 (n = 10/group). (**E**) Western blot analysis showing the phosphorylation levels of Akt in muscle, epididymal adipose tissues and liver tissues, as well as FoxO1, GSK3β and GS in the liver. (**F**) Relative glycogen content in comparison to that of the NC group. Values shown are means ± SD, * *p* < 0.05 vs. NC group; # *p* < 0.05 vs. HFD group.

Next, we examined the effect of SFA on blood glucose using an intraperitoneal glucose tolerance test and insulin tolerance test. The results showed both 0.5 mg/kg and 5 mg/kg SFA markedly decreased blood glucose levels in glucose tolerance test in HFD-fed mice (Figure 5A,B). However, only 5 mg/kg but not 0.5 mg/kg SFA decreased blood glucose levels in insulin tolerance test (Figure 5C,D) and recovered the reduction of glycogen levels (Figure 5F). In addition, we analyzed the phosphorylation of crucial participants in insulin-signaling cascades. We found HFD decreased the phosphorylation of Akt in liver, muscle and epididymal adipose tissues. However, SFA treatment rescued these reductions in HFD-fed mice, indicating that SFA administration could enhance insulin sensitivity not only in liver tissue, but also in muscle and adipose tissues. Furthermore, the in vivo Western Blot results also showed that both 0.5 mg/kg and 5 mg/kg SFA treatments reversed the changes in hepatic p-FoxO1, p-GSK3β and p-GS in insulin signaling transduction pathway in HFD-fed mice (Figure 5E). These data showed that SFA improved insulin sensitivity in HFD-fed mice and 5 mg/kg SFA had a better effect than 0.5 mg/kg SFA.

**Figure 6:**
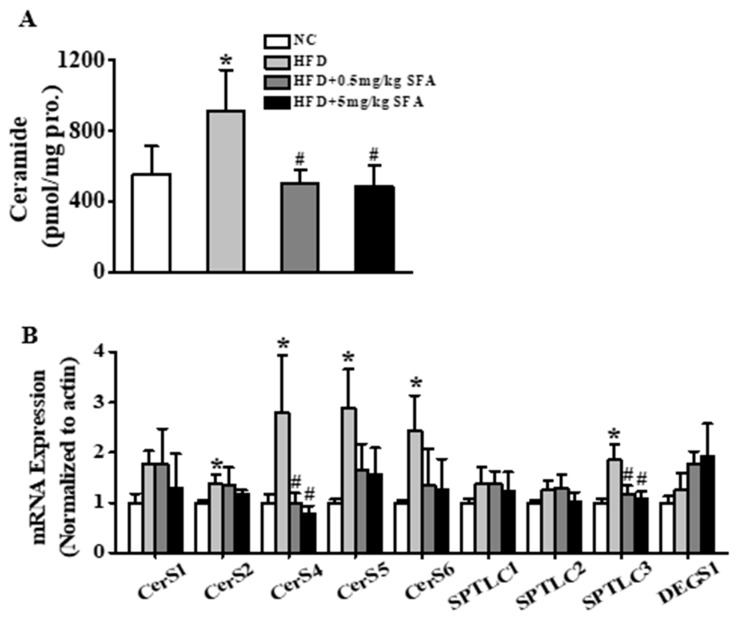
Effects of SFA on ceramide contents and transcription levels of ceramide-related genes in vivo. (**A**) Total ceramide contents were detected by LC/MS/MS. (**B**) The transcription levels of SPTLC1, 2 and 3, CerS1, 2, 4, 5 and 6, and DEGS1 determined by RT-PCR. Values shown are means ± SD for 4 to 6 mice per group, * *p* < 0.05 vs. NC group; # *p* < 0.05 vs. HFD group.

Moreover, mice fed on HFD demonstrated significantly elevated hepatic ceramides compared with mice in the NC group. Treatment with SFA completely normalized ceramide levels such that by the end of the treatment period, hepatic ceramide levels were not different from those in the NC group (Figure 6A and Figure S5). Furthermore, HFD treatment increased transcription level of ceramide-related genes, whereas SFA treatment obviously reversed SPTLC3 and CerS4 expression (Figure 6B). Altogether, these data strongly indicated that SFA improved glucose tolerance and insulin sensitivity, reduced ceramide accumulation and ameliorated ceramide biosynthesis in HFD-fed mice.

## 4. Discussion

As a natural compound in cruciferous vegetables, and for its ability to slow tumor growth, SFA has been studied extensively as a chemotherapeutics for cancer treatment. Interestingly, recent studies in both rodents and humans reveal that SFA has insulin-sensitizing properties, suggesting an alternative use for this compound [21,22]. Because of these studies, clinical trials evaluating the utility of SFA for treating insulin resistance have been initiated. In this study, we demonstrated that SFA alleviated PA-induced insulin resistance by preventing ceramide accumulation. In HFD-fed mice, SFA treatment improved glucose tolerance and insulin sensitivity, inhibited hepatic ceramide production and attenuated hepatic ceramide biosynthesis. Interestingly, SFA also significantly reduced hepatic triglyceride level, suggesting a reversal in hepatic steatosis [33].

Insulin plays an important role in inhibition of gluconeogenesis and stimulation of glycogen synthesis in the liver. The insulin cascade begins with the binding of insulin to its membrane receptor, which leads to tyrosine phosphorylation of IRS-1 with subsequent activation Akt [34]. Akt catalyzes FoxO1 phosphorylation which leads to its nuclear exclusion and downregulation of genes associated with gluconeogenesis [35]. In addition, activation of Akt will lead to an inhibition of GSK3β by phosphorylation, and subsequent dephosphorylation and activation of GS to enhance glycogen synthesis [36]. However, under insulin-resistant conditions, IRS-1 Ser307 site is phosphorylated, which prevents coupling of IRS-1 with insulin receptor. This in turn inhibits downstream signaling pathway to modulate glucose metabolism [36,37]. A previous study shows that astaxanthin treatment promotes the IRS-1/Akt pathway in the livers of insulin-resistant mice via decreasing serine phosphorylation of IRS proteins and improving glucose metabolism [38]. In the current study, we found that SFA improved the impaired glucose metabolism in insulin-resistant HepG2 cells partially through promoting glycogenesis and inhibiting gluconeogenesis. These data show that SFA attenuates insulin resistance and recovers glucose homeostasis by regulating the IRS-1/Akt signaling pathway.

Ceramides are a class of sphingolipids that are found in membrane and function as an intracellular signaling molecules [39,40]. SPTLC, composed of SPTLC1, SPTLC2, and SPTLC3 subunits, is the first key enzyme involved in the rate-limiting step of ceramide biosynthesis. Multiple reports showed that inhibiting SPTLC activity with RNAi [41] or myriocin [42] in rodents or cell lines or by genetic modulation in mice reduces ceramide levels, and subsequently improves lipid profiles and prevents the onset of diabetes [17,43,44]. We herein demonstrated that SFA blocked lipid-induced ceramide accumulation by modulating SPTLC3. Using targeted siRNA against SPTLC3, we were able to confirm the role of SPTLC3 in decreasing ceramide levels and recovering glucose uptake. Moreover, chronic SFA treatment of diet-induced obese mice alleviated insulin resistance and decreased ceramide contents by regulating the expression of ceramide biosynthesis-related gene SPTLC3. In agreement with our results, Kun-Ho Seo et al also confirmed that chardonnay grape seed flour ameliorated hepatic insulin resistance and downregulated ceramide synthesis [45]. Thorsten Hornemann et al. showed that SPTLC3 is highly expressed in human liver tissues and HepG2 cells. Moreover, SPTLC3 overexpression increased SPTLC activity while SPTLC3 silencing resulted in a significant decrease in SPTLC activity [46]. These findings support our results on the role of SPTLC3 in the insulin resistance of liver and HepG2 cells. Collectively, our study revealed a new mechanism underlying SFA’s insulin-sensitizing properties and suggested that therapeutically targeting ceramide synthesis through inhibiting SPTLC3 might be an effective therapeutic for insulin resistance. Interestingly, SFA also reduced hepatic triglyceride, indicating that SFA could reverse hepatic steatosis [47]. It remains to be investigated whether hepatic steatosis is a consequence or a cause of ceramide-mediated insulin resistance.

In conclusion, our study revealed that SFA treatment could recover hepatic glucose homeostasis and improve insulin sensitivity both in vitro and in vivo by suppressing ceramide synthesis. The reduction of ceramide production by SFA is due to downregulation of SPTLC3 expression. To the best of our knowledge, our study is the first to suggest that therapeutically targeting ceramide synthesis through inhibiting SPTLC3 could be an effective treatment for insulin resistance, and SFA may be a potential candidate for prevention and amelioration of hepatic insulin resistance via a ceramide-dependent mechanism. Pharmacologically reducing ceramide contents might also be a novel approach for us to seek more effective bioactive compounds to prevent or treat obesity-induced insulin resistance and T2DM.

## Supplementary Materials

The following are available online at https://www.mdpi.com/2072-6643/11/5/1185/s1, Table S1: siRNA sequences used in this study, Table S2: The primers for RT-PCR, Figure S1: Ceramide contents analyzed by LC/MS/MS in HepG2 cells treated with or without 100 µM PA in the absence or presence of 10 µM SFA for 24 h, followed by 100 nM insulin stimulation for 10 min. Values are presented as means ± SD from three independent experiments. * *p* < 0.05 vs. control cells; # *p* < 0.05 vs. PA-treated cells, Figure S2: (A and D) The transcription level of Cer2 and Cer4 determined by RT-PCR, (B and E) cellular glucose uptake expressed as percentage of mean fluorescence intensity relative to control cells and (C and F) ceramide contents detected by LC/MS/MS in HepG2 cells transfected with control siRNA or Cer2 and Cer4 siRNA, and incubated in medium containing either normal or 100 µM PA for 24 h followed by 100 nM insulin stimulation for 10 min. Values are presented as means ± SD from three independent experiments. * *p* < 0.05 vs. control cells; # *p* < 0.05 vs. scramble group in PA-treated cells, Figure S3: Ceramide contents measured by LC/MS/MS in HepG2 cells transfected with control siRNA or SPTLC3 siRNA and incubated in medium containing either normal or 100 µM PA for 24 h followed by 100 nM insulin stimulation for 10 min. Values are presented as means ± SD from three independent experiments. * *p* < 0.05 vs. scramble group in PA-treated cells, Figure S4: (A) Evaluation of hepatic steatosis after 10 weeks of SFA treatment to HFD-fed mice. a. Images of mouse liver after 10 weeks of different treatments. b. Histological images of liver sections after H&E staining of mice under different treatments in 10 weeks. c. Histological images of liver sections after Oil red O staining of mice under different treatments for 10 weeks. (B) Determination of TG, TC, ALT and AST levels in mouse liver after 10 weeks of different treatments. Values shown are means ± SD for 4 to 6 mice per group, * *p* < 0.05 vs. NC group; # *p* < 0.05 vs. HFD group, Figure S5: Ceramide contents were detected by LC/MS/MS after 10 weeks of SFA treatment for HFD-fed mice. Values shown are means ± SD for 4 to 6 mice per group, * *p* < 0.05 vs. NC group; # *p* < 0.05 vs. HFD group.
