# Supplementary material for: Sulforaphane Prevents Hepatic Insulin Resistance by Blocking Serine Palmitoyltransferase 3-Mediated Ceramide Biosynthesis
Source: Nutrients. 2019 May 27;11(5):1185. doi: 10.3390/nu11051185 (PMC6566605; doi:10.3390/nu11051185)
Supplement: Supplementary file 1 [file nutrients-11-01185-s001.zip › Supplementary data-figure S1-S5.docx]

**Supplementary data：**


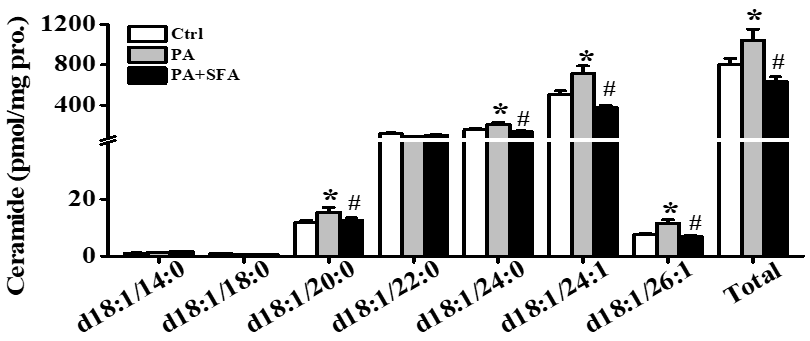


**Figure. S1** Ceramide contents analyzed by LC/MS/MS in HepG2 cells treated with or without 100 µM PA in the absence or presence of 10 µM SFA for 24 h, followed by 100 nM insulin stimulation for 10 min. Values are presented as means ± SD from three independent experiments. **p* < 0.05 *vs*. control cells; # *p <* 0.05 *vs*. PA-treated cells.


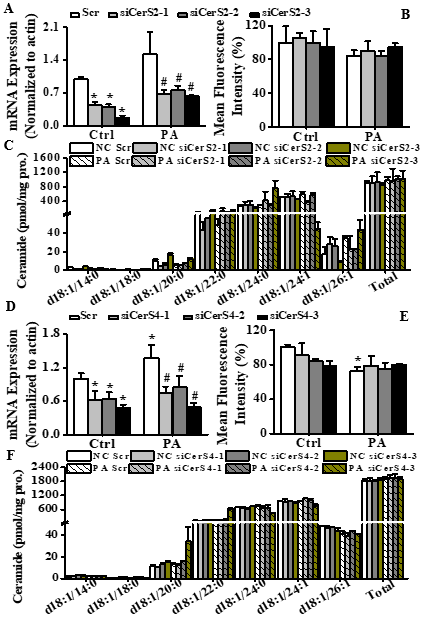


**Figure S2 (A and D)** The transcription level of Cer2 and Cer4 determined by RT-PCR, **(B and E)** cellular glucose uptake expressed as percentage of mean fluorescence intensity relative to control cells and **(C and F)** ceramide contents detected by LC/MS/MS in HepG2 cells transfected with control siRNA or Cer2 and Cer4 siRNA, and incubated in medium containing either normal or 100 µM PA for 24 h followed by 100 nM insulin stimulation for 10 min. Values are presented as means ± SD from three independent experiments. **p* < 0.05 *vs*. control cells; #*p* < 0.05 *vs*. scramble group in PA-treated cells.


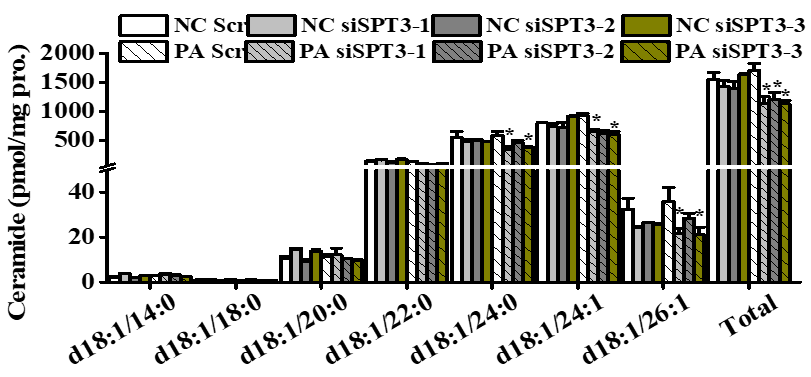


**Figure S3** Ceramide contents measured by LC/MS/MS in HepG2 cells transfected with control siRNA or SPTLC3 siRNA and incubated in medium containing either normal or 100 µM PA for 24 h followed by 100 nM insulin stimulation for 10 min. Values are presented as means ± SD from three independent experiments. **p* < 0.05 *vs*. scramble group in PA-treated cells.


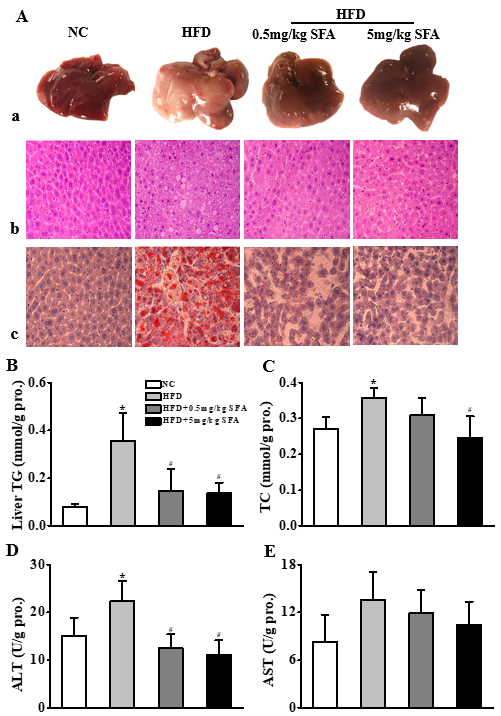


**Figure S4 (A)** Evaluation of hepatic steatosis after 10 weeks of SFA treatment to HFD-fed mice. **a.** Images of mouse liver after 10 weeks of different treatments. **b.** Histological images of liver sections after H&E staining of mice under different treatments in 10 weeks. **c.** Histological images of liver sections after Oil red O staining of mice under different treatments for 10 weeks. **(B)** Determination of TG, TC, ALT and AST levels in mouse liver after 10 weeks of different treatments. Values shown are means ± SD for 4 to 6 mice per group, **p* < 0.05 vs. NC group; # *p <* 0.05 vs. HFD group.


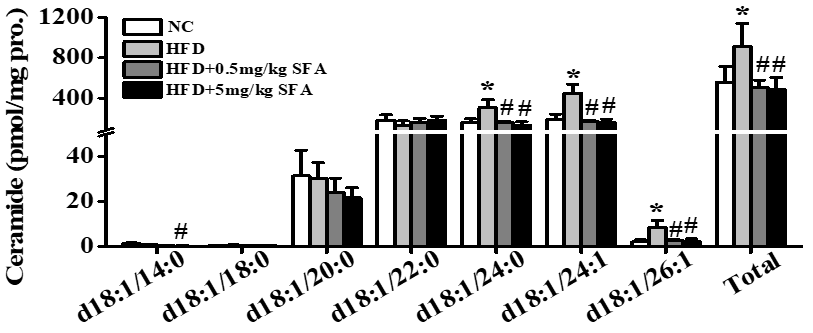


**Figure S5** Ceramide contents were detected by LC/MS/MS after 10 weeks of SFA treatment for HFD-fed mice. Values shown are means ± SD for 4 to 6 mice per group, **p* < 0.05 vs. NC group; # *p <* 0.05 vs. HFD group.
