# Supplementary material for: Sulforaphane Prevents Hepatic Insulin Resistance by Blocking Serine Palmitoyltransferase 3-Mediated Ceramide Biosynthesis
Source: Nutrients. 2019 May 27;11(5):1185. doi: 10.3390/nu11051185 (PMC6566605; doi:10.3390/nu11051185)
Supplement: Supplementary file 1 [file nutrients-11-01185-s001.zip › Supplementaty Table.docx]

**Supplementary Table S1**. siRNA sequences used in this study

|  | Sense (5'→3') | Antisense (5'→3') |
| --- | --- | --- |
| SPTLC3-1 | CCUCGAACCCGCAGAGCUUTT | AAGCUCUGCGGGUUCGAGGTT |
| SPTLC3-2 | GCACCUUGGAUAAGCACAATT | UUGUGCUUAUCCAAGGUGCTT |
| SPTLC3-3 | CCAAGUAUGAUGAGUCUAUTT | AUAGACUCAUCAUACUUGGTT |
| CerS2-2 | GGAUAAACCCUGGUUCUAUTT | AUAGAACCAGGGUUUAUCCTT |
| CerS2-2 | GCACUAUCCCUUCCCAGUATT | UACUGGGAAGGGAUAGUGCTT |
| CerS2-3 | GCAUGGCCCACAAGUUCAUTT | AUGAACUUGUGGGCCAUGCTT |
| CerS4-1 | GCCUUGCCUUUGAGAGAUUTT | AAUCUCUCAAAGGCAAGGCTT |
| CerS4-2 | GCAGGACAGGUUCUGGUUATT | UAACCAGAACCUGUCCUGCTT |
| CerS4-3 | GGUCAACUACAUGCAGUAUTT | AUACUGCAUGUAGUUGACCTT |
|  |  |  |

**Supplementary Table S2**. The primers for RT-PCR.

|  | Forward primer (5'→3') | Reverse primer (5'→3') |
| --- | --- | --- |
| Human: |  |  |
| β-actin | CATGTACGTTGCTATCCAGGC | CTCCTTAATGTCACGCACGAT |
| CerS1 | CACTGCGCGCCTCTTTCG | ATTGTGGTACCGGAAGGCG |
| CerS2 | GCTGGAGATTCACATTTTAC | GAAGACGATGAAGATGTTGT |
| CerS3 | GCTATATGACTTATGGGAGG | AAGATCATGAGCTGTAGGTT |
| CerS4 | GTTTCAACGAGTGGTTTTG | TGAATCTCTCAAAGGCAAG |
| CerS5 | ATCTTCTTCGTGAGGCTG | ATGTCCCAGAACCAAGGT |
| CerS6 | ATCAGGAGAAGCCAAGCACG | AGTAGTGAAGGTCAGTTGTG |
| SPTLC1 | AAGAAGCCATTATATACTCATAT | GGCACTGATAAGATCAATA |
| SPTLC2 | GAGTGTGTACAACAGTTAGCTG | TGGCTCACAAAGGCCAC |
| SPT3LC | TGCAGCCAAGTATGATGAGTCTA | GCAGATGCACGATGGAAC |
| DEGS1 | TTCTTCTGTACCGCTTTCAG | TTACTCCAGCACCATCTCT |
| Mouse: |  |  |
| β-actin | GTGACGTTGACATCCGTAAAGA | GCCGGACTCATCGTACTCC |
| CerS1 | GGTCAGATGCGTGAACTGGAA | GGATAGAGTCCTGGATGGCTGAA |
| CerS2 | CGTGTCTATGCCAAAGCCTCA | GTCTGGTAGAAATGTTCCAAGGTG |
| CerS4 | CATGACTGCTCCGACTACCTG | GAATATGAGGCGCGTGTAGAA |
| CerS5 | ACGTGAGCGGCTCTGTACCA | GAGCACCTGCAGGATCAGGA |
| CerS6 | CACCTGGGCAGACCTGAAGA | TGGCACATGGTTTGGCTATGA |
| SPTLC1 | TCGAGTTAAGGCCACAGCTT | CATAGAACCCTCGAGGACCA |
| SPTLC2 | GGATACATCGGAGGCAAGAA | GACATCGACGTGGCATACAC |
| SPTLC3 | CCGCTAAAGTGTCTGCTTT | CTTCAAAGCTTGCTTCATTG |
| DEGS1 | ATGTCTTTGGCAGCTGCCTT | CATTCCAAACCAGCGGTTCC |
